# Quantitative analysis of commensal *Escherichia coli* populations reveals host-specific enterotypes at the intra-species level

**DOI:** 10.1002/mbo3.266

**Published:** 2015-05-29

**Authors:** Mounira Smati, Olivier Clermont, Alexandre Bleibtreu, Frédéric Fourreau, Anthony David, Anne-Sophie Daubié, Cécile Hignard, Odile Loison, Bertrand Picard, Erick Denamur

**Affiliations:** 1 INSERM, IAME, UMR 1137F-75018, Paris, France; 2 Univ Paris Nord, IAME, UMR 1137Sorbonne Paris Cité, Paris, France; 3 APHP, Hôpitaux Universitaires Paris Seine Saint-Denis, Hôpital Avicenne, Service de Bactériologie-Virologie-HygièneBobigny, France; 4 Univ Paris Diderot, IAME, UMR 1137Sorbonne Paris Cité, Paris, France; 5 Univ Paris Diderot, Station d’Ecologie ForestièreSorbonne Paris Cité, Fontainebleau, France

**Keywords:** Commensal, enterotypes, *Escherichia coli*, quantitative analysis, microbiota

## Abstract

The primary habitat of the *Escherichia coli* species is the gut of warm-blooded vertebrates. The *E. coli* species is structured into four main phylogenetic groups A, B1, B2, and D. We estimated the relative proportions of these phylogroups in the feces of 137 wild and domesticated animals with various diets living in the Ile de France (Paris) region by real-time PCR. We distinguished three main clusters characterized by a particular abundance of two or more phylogroups within the *E. coli* animal commensal populations, which we called “enterocolitypes” by analogy with the enterotypes defined in the human gut microbiota at the genus level. These enterocolitypes were characterized by a dominant (>50%) B2, B1, or A phylogroup and were associated with different host species, diets, and habitats: wild and herbivorous species (wild rabbits and deer), domesticated herbivorous species (domesticated rabbits, horses, sheep, and cows), and omnivorous species (boar, pigs, and chickens), respectively. By analyzing retrospectively the data obtained using the same approach from 98 healthy humans living in Ile de France (Smati et al. 2013, *Appl. Environ. Microbiol*. 79, 5005–5012), we identified a specific human enterocolitype characterized by the dominant and/or exclusive (>90%) presence of phylogroup B2. We then compared B2 strains isolated from animals and humans, and revealed that human and animal strains differ regarding O-type and B2 subgroup. Moreover, two genes, *sfa/foc* and *clbQ*, were associated with the exclusive character of strains, observed only in humans. In conclusion, a complex network of interactions exists at several levels (genus and intra-species) within the intestinal microbiota.

## Introduction

Cells of the gut microbiota in vertebrates often outnumber those of the host, and are required for development as well as homeostasis in adult life. The gut microbiota performs important functions, such as fermentation of nondigestible dietary components in the large intestine, and maintains balance between the host’s metabolism and immune system (Flint 2012). The bacterial communities of the microbiota codiversified with their hosts and their composition depend on several factors including host phylogeny, gut physiology, and host diet (Ley et al. 2008; Muegge et al. 2011; Wu et al. 2011; David et al. 2014). Humans living a modern lifestyle have a gut microbiota that is typical of omnivorous primates. Recently, three main clusters called enterotypes, corresponding to distinct structures with the bacterial community, were identified in the human gut microbiome (Arumugam et al. 2011). These enterotypes correspond to clusters containing a particular abundance of different phyla and genera. These three enterotypes have also been found in chimpanzees, supporting the hypothesis that enterotypic variation was present in great apes before the divergence of humans and chimpanzees (Moeller et al. 2012). Strict anaerobes constitute most of this microbiota; however, it also contains aerobes, including *E. coli*, which is the pre-dominant aerobic organism in the gastrointestinal tract. *Escherichia coli* inhabits the intestines and feces of warm-blooded animals and reptiles (Gordon and Cowling 2003). In humans, it is found at around 10^8^ colony forming units (CFU) per gram of feces. Thus, in 16S rRNA analyses of the microbiota and whole-genome shotgun sequence analyses of the microbiome, the *Escherichia* genus is at the threshold of detection (Arumugam et al. 2011; Claesson et al. 2012).

*E. coli* is most frequently found in humans (more than 90% of humans are colonized by this bacterium) (Lescat et al. 2013; Smati et al. 2013) followed by domesticated (64%) and wild (45%) animals (Gordon and Cowling 2003; Lescat et al. 2013). *E. coli* is also more frequent in mammals (56%) than in birds (23%) and reptiles (10%) (Gordon and Cowling 2003; Tenaillon et al. 2010). Studies analyzing the intraspecific genetic structure of the *E. coli* species have shown that it is a clonal species (Desjardins et al. 1995) comprised of at least four main phylogenetic groups designated A, B1, B2, and D (Ochman and Selander 1984; Herzer et al. 1990). Interestingly, the commensal ecological niches of these groups are different. Commensal human strains in developed countries belong mainly to the B2 phylogenetic group (43%), followed by the A (24%), D (21%), and B1 (12%) phylogroups (Tenaillon et al. 2010). Domesticated animals have a higher ratio of A to B2 strains than their wild counterparts (Escobar-Páramo et al. 2006; Baldy-Chudzik et al. 2008). Furthermore, factors that shape the overall composition of the microbiota such as gut anatomy, physiology, and diet also influence colonization by *E. coli* and its various phylogenetic groups (Gordon and Cowling 2003; Tenaillon et al. 2010).

However, most epidemiological studies of *E. coli* commensal strains have involved only one (considered the dominant) or a few clones. Using a real-time PCR method to quantify both the total number of *E. coli* and that of the four main phylogenetic group strains, we recently reported that within-individual diversity of the various phylogenetic groups is high in healthy humans. We identified two distinct population structures describing the relative abundance of phylogroups: the dominance of phylogenetic group B2, which excluded the coexistence of other phylogroups in feces, and the dominance of phylogroup A, which was associated with more diversity (Smati et al. 2013). The B2 phylogroup is particularly interesting because strains from this phylogroup are increasingly being found as commensal bacteria in humans living in developed countries (Duriez 2001 et al.; Escobar-Páramo et al. 2004; Tenaillon et al. 2010) and are involved in extraintestinal infections (Picard et al. 1999). This virulence is linked to numerous factors such as adhesins, iron capture systems, toxins, and protectins (Johnson and Stell 2000). These factors have also been reported in commensal strains (Nowrouzian et al. 2005), suggesting that extraintestinal virulence is a by-product of commensalism (Adiba et al. 2010; Diard et al. 2010). Furthermore, group B2 strains harbour a genomic island called “*pks*” that codes for a polyketide-peptide genotoxin, called colibactin (Nougayrède et al. 2006). Colonization of the colon by *E. coli* strains harbouring the *pks* island may contribute to the development of sporadic colorectal cancer (Cuevas-Ramos et al. 2010).

Very few studies have assessed quantitatively the commensal *E. coli* microbiota in animals. Here, we determined by real-time PCR total counts of *E. coli* as well as the proportions of the major *E. coli* phylogenetic groups in the feces of various healthy animal species chosen for their distinct habitat, diet and proximity to humans. In particular, we focused on B2 phylogenetic strains of a subset of animals and compared these strains to those found in humans.

## Materials and Methods

### *Escherichia coli* isolation and identification

We performed sampling between October 2011–February 2012 in the Fontainebleau Forest, Ile de France, France, and in different farms or equestrian clubs of the same region. Depending on the host species being sampled, samples were collected from freshly deposited feces (domesticated animals and wild rabbits) or directly from fecal material in the rectum of hunted animals just after death (deer and boars during species management programs organized by the “Office National des Forêts” in Fontainebleau forest). All animals, except for wild rabbits, were physically examined to ensure that they were healthy. Samples were obtained from 90 domesticated animals (15 pigs, 15 horses, 15 sheep, 15 cows, 15 rabbits, and 15 chickens) and 47 wild animals (15 wild rabbits, 15 boars, and 17 deer). The horses originated form three distinct equestrian clubs, whereas the farm animals originated from one farm for each species. Feces were collected using a swab/transport tube system containing Amies transport medium. The collected material was removed from swabs in glycerol stock solution (Cryobank; Biovalley, Marne La Vallée, France) and stored at −80°C until further use for DNA extraction. The stool-containing suspensions were plated on chromogenic plates (UriSelect®; BioRad, Marnes la Coquette, France). Pink colonies (one per animal) were randomly picked, tested for indole production, identified as *E. coli* if positive (according to the manufacturer’s recommendations) and stored in glycerol stock solution. Fifteen humans from the Coliville study (Smati et al. 2013) were randomly selected using a table of random numbers (Kendall and Babington Smith 1939) and studied in parallel. If a dominant A phylogroup was detected by qPCR, stools were plated onto Drigalski agar plates and lactose negative colonies were sampled. *Escherichia fergusonii* strains were identified by the API20E detection system (BioMérieux, Marcy l’Etoile, France), whereas *Escherichia albertii* were identified by allele-specific PCR with the following primers 5′-TTATCGACTCTTCTACTCCC-3′ and 5′-CACGCATGTTCACATCCTGG-3′ targeting the *chuA* gene (amplification of a 146 bp fragment using standard conditions as described in Clermont et al. 2014).

### Quantitative PCR assay

#### DNA extraction from feces

The QIAamp DNA Stool Minikit (Qiagen, Courtabœuf, France) was used to extract DNA from 200 mg of frozen stool sample according to the manufacturer’s recommendations with some modifications (Smati et al. 2013). The DNA was eluted in a final volume of 200 *μ*L and stored at −80°C.

#### Quantitative PCR assay

A real-time PCR assay targeting the genes amplified by the classical phylogrouping triplex PCR method (Clermont et al. 2000) was used to quantify directly the four main *E. coli* phylogroups (A, B1, B2, and D) from feces. In this assay, the B1 (*chuA* negative, *yjaA* negative, and TspE4.C2 positive), B2 (*chuA* positive, *yjaA* positive, and TspE4.C2 variable), and D phylogroups (*chuA* positive, *yjaA* negative, and TspE4.C2 variable) were quantified by real-time PCR using probes specifically targeting B1 TspE4.C2, B2 *chuA*, and D *chuA* sequences (Smati et al. 2013). The proportion of each phylogroup was calculated as a percentage of the total *E. coli* count obtained by quantitative PCR for 16S rDNA (Penders et al. 2006). The proportion of phylogroup A, which comprises the A_0_ (*chuA*, *yjaA*, and TspE4.C2 negative) and A_1_ (*chuA* negative, *yjaA* positive, and TspE4.C2 negative) subgroups was calculated by subtracting the proportions of phylogroups B1, B2, and D from the total *E. coli* count. The proportion of the A_1_ subgroup was confirmed with a *yjaA-*specific probe (pyjaAA1/B2), which quantifies the A_1_ subgroup and the B2 phylogroup using the following formula: A_1_ = *yjaA* positive – B2. The threshold of phylogroup A detection by subtraction is 15% of the total *E. coli* count. The threshold of detection of B1, B2, and D phylogroups by specific probes is 0.1% of the *E. coli* population (Smati et al. 2013).

### Genotypic characterization of *E. coli* colonies and colicin production

In the validation assay, the phylogenetic group of each randomly picked *E. coli* strain was determined by the quadruplex PCR method (Clermont et al. 2013), which detects seven phylogroups (A, B1, B2, C, D, E, F) of *E. coli* sensu stricto and *Escherichia* clades. Furthermore, *Escherichia* clades were assigned by PCR as described in (Clermont et al. 2011a).

A subset of *E. coli* B2 strains was further subtyped by examining the sequence type complex (STc) by allele-specific PCR as previously described (Clermont et al. 2014), and was also O-typed by PCR as described in (Clermont et al. 2007). Strains were also tested for the presence of 20 major factors of *E. coli* extraintestinal virulence including adhesins (*papC*, *papG*, including *papG* alleles, *sfa/foc*, *iha*, *hra*, and *ibeA*), iron capture systems (*fyuA*, *irp2*, *iroN*, *iucC*, and *ireA*), protectins (*kpsE*, *neuC*, chromosomal *ompT*, and *traT*), and toxins (*hlyC*, *cnf1*, *usp, sat*, and *clbQ*) (Johnson et al. 2008; Clermont et al. 2011b; Lefort et al. 2011). For each isolate, a virulence score, defined as the number of virulence factors present of the 20 tested, was calculated (adapted from Lefort et al. 2011).

The production of colicins and phages by B2 strains was detected by plaque lysis assays, in which *E. coli* K-12 (phylogroup A) was used as the sensitive strain (Riley and Gordon 1992; Gordon and O’Brien 2006). Briefly, 10 *μ*L of an overnight (O/N) culture of each strain grown in lysogeny broth (LB) medium was spotted onto LB agar containing mitomycin (25 *μ*mol/L) onto which an O/N culture of the *E. coli* K-12 strain diluted to 0.5 McFarland units had already been plated. After O/N culture at 37°C, the result of the assay was considered to be positive if the strain was surrounded by a halo, corresponding to inhibition of the growth of the sensitive strain (adapted from Bleibtreu et al. 2013).

### Statistical analyses

Factorial analysis of correspondence (FAC) was used to describe associations between groups and two-way tables were analyzed with SPAD.N software (Cisia, Saint-Mandé, France). Two tables were used. The first table had 132 rows corresponding to the *E. coli* strains from stool samples of 132 animals and 32 columns, corresponding to the 32 variables. The 32 variables were the dominant phylogenetic groups (A, B1, B2, or D), the intermediate phylogenetic groups, the minor phylogenetic groups, and the absence of a particular phylogenetic group (see definitions below) as determined by qPCR; the presence of a high (more than 10^7^ CFU per gram), intermediate (between 10^6^ and 10^7^ CFU per gram), or low (less than 10^6^ CFU per gram) quantity of *E. coli* in feces, high genetic diversity (corresponding to four distinct phylogenetic groups per individual) or low genetic diversity (corresponding to three or less distinct phylogenetic groups per individual), the animal species (horse, cow, sheep, pig, wild rabbit, domesticated rabbit, deer, boar, chicken); and the animal’s habitat (wild and domesticated) and diet (herbivorous, omnivorous).

The second table had 43 rows corresponding to 43 phylogenetic group B2 strains (30 strains of human origin and 13 strains of animal origin) and 33 columns (corresponding to the 33 variables). The 33 variables were human origin and “exclusive” group B2, human origin and “non-exclusive” group B2, animal origin, the eight B2 subgroups, the unassignable B2 subgroup, the 20 virulence factor genes (see Table3), and a virulence score of 9 or more.

A binary code was used for each variable: present, 1; absent, 0. FAC is an eigenvector method of ordination that uses a covariance matrix based on chi-square distances. It describes the dispersion and shape of a cloud of *n* objects (here, the *E. coli* strains) or *P* variables (here, the studied variables) in a multidimensional space, by replacing the original data set by a new set of orthogonal linear coordinates in a space of significantly lower dimension. The explained variances of the elements of the data set (the strains and the variables) are in decreasing order of magnitude with respect to these new coordinates. The variables used for FAC are categories. The computation determines a plane defined by the first two principal axes of the analysis; the first axis, F1, accounts for most of the variance, and the second axis F2, orthogonal to F1, accounts for the largest part of the variance that is not accounted for by F1 (Greenacre 1992). Significance was assessed with a chi-square test with a threshold of *P *<* *0.05.

CFU counts of the various phylogroups were compared with the Wilcoxon or Kruskal–Wallis test depending on the number of classes of tested variables. All tests were two-tailed and a significance level of 0.05 was used. All statistical analyses were carried out with R software (R Development Core Team, 2008).

## Results

### Characteristics of the studied populations

During this study, we established a collection of animal feces from the Ile de France region. In total, 137 fresh animal feces were collected, either from material deposited on the ground or collected directly from the rectum. Among these animals, 90 were domesticated (15 pigs, 15 horses, 15 sheep, 15 cows, 15 rabbits, and 15 chickens) and 47 were wild (15 wild rabbits, 15 boars, and 17 deer). Thus, we sampled 92 herbivorous animals (sheep, horses, cows, rabbits, and deer) and 45 omnivorous animals (chicken, boars, and pigs).

### Prevalence and quantification of *E. coli* in stools of domesticated and wild animals compared to humans

We first quantified the total amount of *E. coli* in the feces of the 137 studied animals by qPCR as described previously (Penders et al. 2006). The prevalence of *E. coli*, defined as the proportion of hosts colonized by *E. coli*, was 100% in all species except domesticated rabbits (66%) (Table1).

**Table 1:**
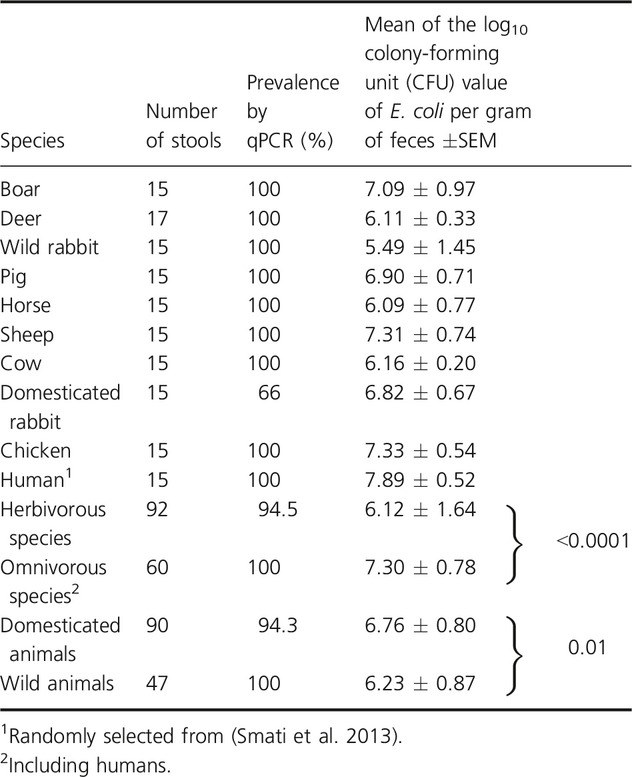
Prevalence and quantity of *Escherichia coli* in feces of domesticated animals, wild animals, and humans

Counts of *E. coli* varied between species (Table1). Wild rabbits had the lowest counts of *E. coli* (5.49 ± 1.45 log CFU of *E. coli* per gram of feces), whereas humans had the highest (7.89 ± 0.52 log CFU of *E. coli* per gram of feces). Diet had a very strong effect on total counts of *E. coli*, with omnivores and herbivorous showing 7.30 and 6.12 log CFU of *E. coli* per gram of feces, respectively (*P *<* *0.0001). This effect was still present when humans were excluded from the analysis, with omnivores and herbivores now showing 7.11 and 6.12 log CFU of *E. coli* per gram of feces, respectively (*P* < 0.0001). The effect of domestication was substantially lower than that of diet, albeit significant (*P *=* *0.01), with domesticated and wild animals showing 6.76 and 6.23 log CFU of *E. coli* per gram of feces, respectively).

### Validation of the qPCR method for animal feces

We recently described a qPCR assay to determine the respective proportions of the four main phylogenetic groups A, B1, B2, and D in stools (Smati et al. 2013). We previously showed that the dominant phylogenetic group determined by qPCR on one randomly selected clone is identical to that determined by classical triplex PCR (Clermont et al. 2000) in 78% of stool samples isolated from healthy humans, with randomization mainly accounting for any discrepancies between the two techniques. However, the diversity of *E. coli* in animal feces differs from that in humans; for example, more strains belong to E phylogroups (Clermont et al. 2011b) or *Escherichia* clades in animals than in humans (Walk et al. 2009; Clermont et al. 2011a). These strains are identified by our qPCR as D (E phylogroup *E. coli*) or A or B2 (*Escherichia* clades) (Smati et al. 2013). Furthermore, *E. fergusonii* and *E. albertii,* both of which are lactose-negative, have been reported in animals (Herráez et al. 2005; Hariharan et al. 2007; Oh et al. 2011). Our qPCR assay identifies these strains as belonging to the A phylogroup (Smati et al. 2013).

To validate our assay on animal stools, we randomly selected one clone on UriSelect plates per animal, which we subsequently labeled the dominant clone. We then studied this clone by the quadruplex PCR method, which divides strains into the seven main *E. coli* phylogenetic groups (A, B1, B2, C, D, E, and F) (Clermont et al. 2013), and by allele-specific PCR to determine the *Escherichia* clade (Clermont et al. 2011a) (data not shown). The E and F phylogroups were found in both wild and domesticated animals (12 strains, 9.1% of isolates) and were assigned as D (Clermont et al. 2000). The C phylogroup strains (eight strains corresponding to 6% of isolates, including 50% of strains found in pigs) were assigned as A (Clermont et al. 2000). Four strains, phenotypically indistinguishable from *E. coli*, were also found. Three were detected in boars and deer and belonged to *Escherichia* clades III and V and one was detected in chickens and belonged to *Escherichia* clade I (Walk et al. 2009; Clermont et al. 2011a). For the 26 samples containing predominantly A phylogroup strains, we also studied lactose-negative colonies (three per plate if present), and identified *E. fergusonii* and *E. albertii* strains by API20E and *chuA* allele-specific PCR, respectively. We detected only one strain of *E. fergusonii*, which was found in sheep, and no strain of *E. albertii*.

Collectively, we found 72% of concordance between the qPCR assay and the triplex PCR method on one randomly selected clone, consistent with results in humans (Smati et al. 2013). The phylogroups C, E, and F, which represent less than 15% of the strains, as well as non-*E. coli Escherichia* (five strains/132 samples = 3.8%), should not affect the overall results of the qPCR assay.

### Prevalence and diversity of *E. coli* phylogroups: definition of the “enterocolitypes”

One advantage of the qPCR assay over plating is its ability to detect minor clones (Smati et al. 2013). With this method, we found that the prevalence of the four phylogenetic groups varied from 6.7% to 100% depending on the host species (Table2). Two, three, or four phylogenetic groups were detected in some animal species, but no unique phylogroup was found. This result contrasts with our previous study in humans, where we found that 21% of healthy humans were colonized by a unique phylogroup (Smati et al. 2013). The main factor affecting diversity was domestication; 55.4% of domesticated animals carried the four main phylogroups versus only 10.6% of wild animals (*P *<* *0.001). In addition, the B1 and D phylogroups were also more frequently detected in domesticated than in wild animals (95.3% in domesticated animals versus 29.8% in wild animals for the B1 phylogroup, and 74.1% versus 46.8% for the D phylogroup, *P *<* *0.001) (Table2). The prevalence of B2 and A phylogroups was similar between domesticated and wild animals and diet did not appear to affect diversity.

**Table 2:**
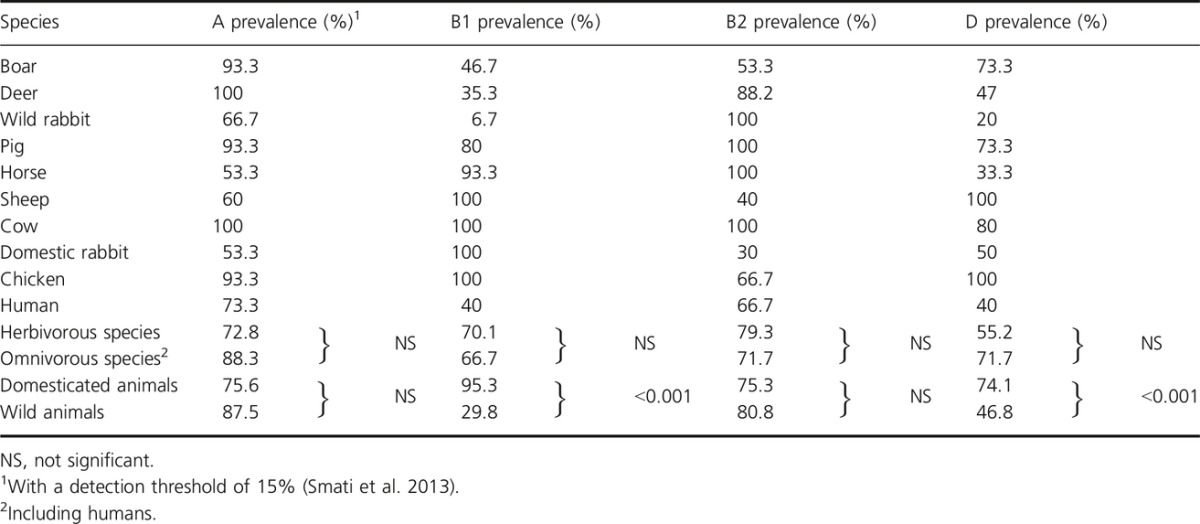
Prevalence of *Escherichia coli* A, B1, B2, and D phylogroups in the feces of the studied species as determined by qPCR

**Figure 1:**
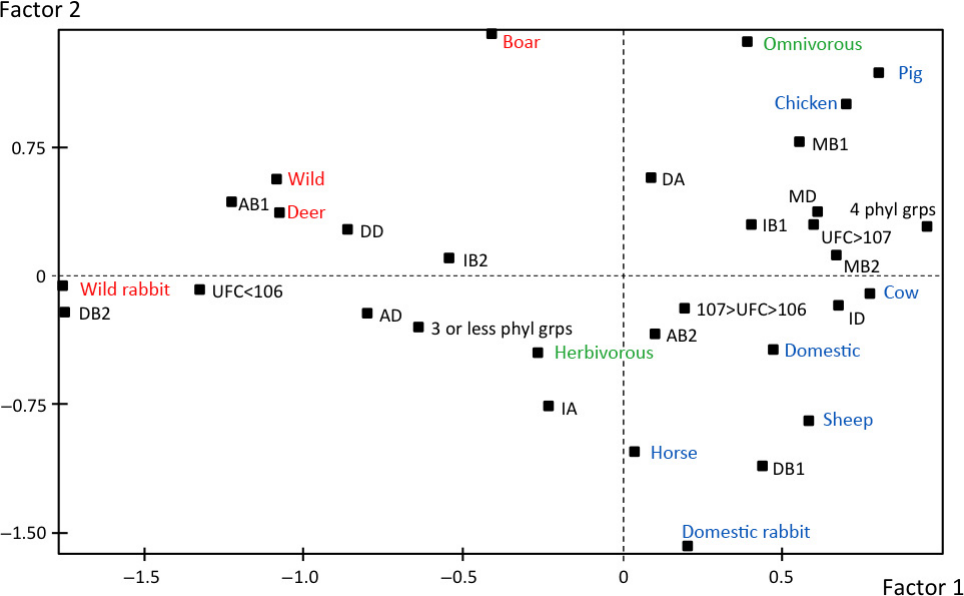
Factorial analysis of correspondence for the 132 animals with stool samples containing *Escherichia coli*. Projections on the F1–F2 plane of the dominant phylogenetic groups (DA, DB1, DB2 and DD), the intermediate phylogenetic groups (IA, IB1, IB2 and ID), the minor phylogenetic groups (MB1, MB2, and MD), of the absence of the phylogenetic groups (AB1, AB2, and AD), high genetic diversity (four phyl grps), low genetic diversity (three or less phyl grps), counts of *E. coli* per gram of feces (CFU > 10^7^, 10^7^ < CFU < 10^6^, and CFU < 10^6^), the animal species (horse, cow, sheep, wild rabbit, domesticated rabbit, deer, pig, boar, and chicken), habitat (wild and domesticated), and diet (herbivorous and omnivorous). The bacterial characteristics appear in black, the animal species of wild and domestic origin are in red and blue, respectively, and the diets are in green.

We divided the phylogroups into four categories adapted from (Schlager et al. 2002) (dominant phylogenetic group: >50% of the population of *E. coli*; intermediate phylogenetic group: 10–50% and minor phylogenetic group <10%; absent phylogenetic group: undetectable), and found that the proportions of these categories varied significantly depending on the phylogenetic group and the host. This variation was complex and we described it by a FAC (Fig.1). On the F1-F2 plane, which accounted for 31.43% of the total variance, the negative value of the first factor distinguished a cluster of variables: the dominant phylogenetic group B2, the absence of the B1 phylogenetic group, the dominant phylogenetic group D, and the absence of this group, the intermediate phylogenetic group B2, the intermediate phylogenetic group A, the deer and wild rabbit species, a wild habitat, the herbivorous diet, a low quantity of *E. coli* in stools (<10^6^ CFU), and a low genetic diversity (three or fewer phylogenetic groups). The positive values of the first factor distinguished several other variables, which could be separated into two main clusters by the positive or negative values of the second factor. For the negative values of the second factor, these variables were the dominant phylogenetic group B1, the absence of phylogenetic group B2, the intermediate phylogenetic group D, the horse, cow, sheep, and domesticated rabbit species, a domesticated habitat and an intermediate quantity of *E. coli* in stools (between 10^6^ and 10^7^ CFU). For the positive values of the second factor, the variables were the dominant phylogenetic group A, the intermediate phylogenetic group B1, the minor phylogenetic groups B1, B2, and D, the pig and chicken species, an omnivorous diet, a high quantity of *E. coli* in stools (>10^7^ CFU) and high genetic diversity (four phylogenetic groups). The variable “boar species” was projected on an intermediate position between the first and the third clusters of the analysis.

**Figure 2:**
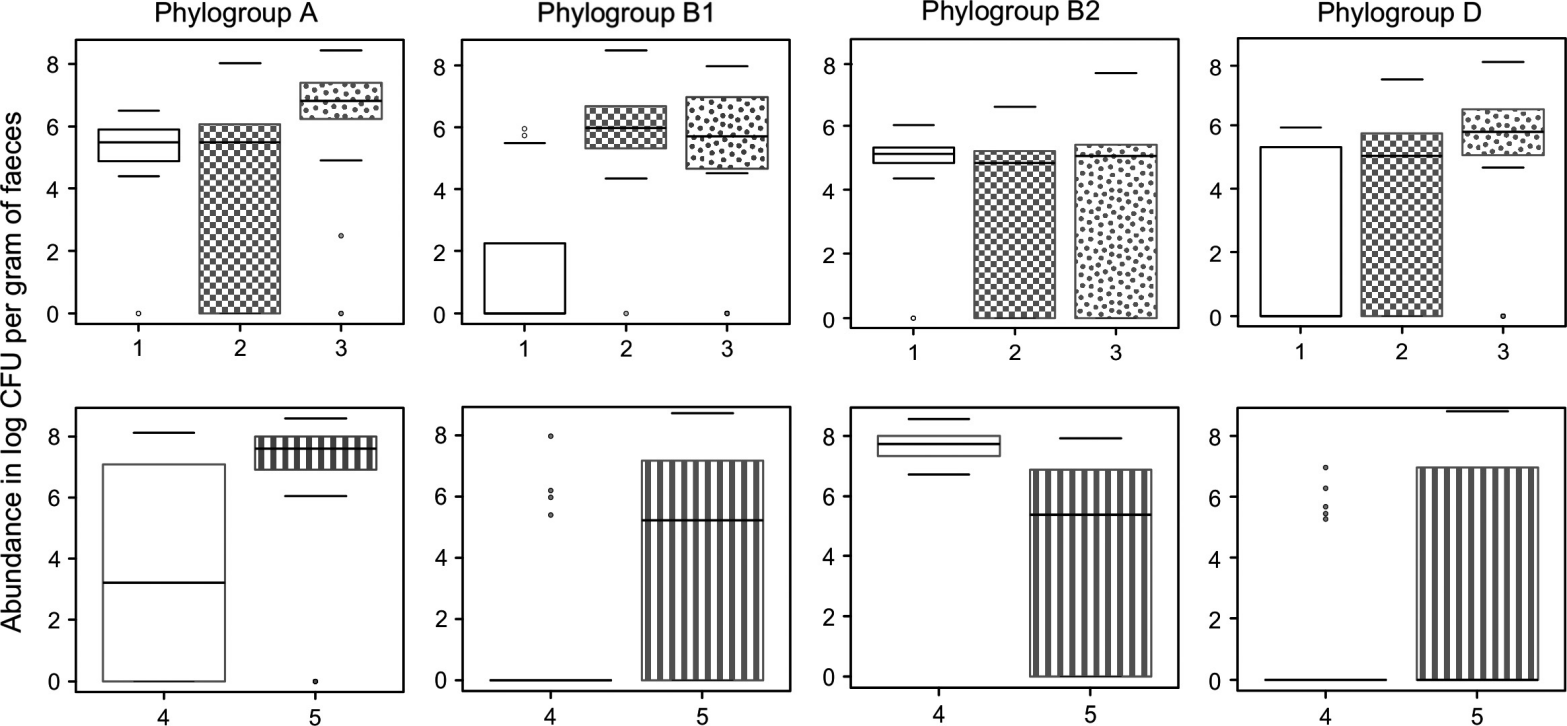
Relative abundance of the *Escherichia coli* strains from the four main phylogroups (A, B1, B2, D) in each “enterocolitype” of animals (upper panel) and humans (lower panel). Enterocolitype 1 (wild rabbits and deer), enterocolitype 2 (domesticated rabbits, horses, sheep, cows), enterocolitype 3 (boar, pigs, chicken), enterocolitype 4 (human B2 “exclusive”), and enterocolitype 5 (human A dominant). Enterocolitypes 4 and 5 are based on data obtained from (Smati et al. 2013). The results are presented as box plots showing the distribution of the estimated CFU per gram of feces. The black bars within each box plot show the median value. The upper and lower limits of the box correspond to the upper and lower quartiles, respectively. Bars above and below the box correspond to 1.5 times the interquartile range. Dots located at some distance outside the box correspond to outliers lying more than 1.5 times beyond the interquartile range.

The FAC analysis thus distinguished three main clusters of *E. coli* animal commensal populations differing in terms of the relative abundance of phylogroup strains that they contain. By analogy with the enterotypes defined at the genus level in the gut microbiota of humans (Arumugam et al. 2011), we propose the delineation of three “enterocolitypes” based on the FAC. Enterocolitype 1 is characterized by low counts of *E. coli*, dominance of the phylogenetic group B2, and low phylogenetic diversity. This enterocolitype is associated with wild and herbivorous animal species (wild rabbits and deer). Enterocolitype 2 is characterized by intermediate counts of *E. coli* and dominance of phylogenetic group B1, and is associated with the domesticated herbivorous animal species (domesticated rabbits, horses, sheep, and cows). Enterocolitype 3 is characterized by high counts of *E. coli* and dominance of phylogroup A associated with variable proportions of other groups, resulting in high phylogenetic diversity. This enterotype is associated with omnivorous species (boar, pigs, and chickens). The abundance of the strains from the phylogenetic groups A, B1, and D in these three enterocolitypes is significantly different (*P *<* *0.001) (Fig.2, upper panel).

In light of these results, we used the same approach to analyse retrospectively data from our previous study involving 98 healthy humans (Smati et al. 2013). We propose the delineation of two additional human enteocolitypes, both characterized by high counts of *E. coli* (7.84 ± 0.54 log CFU of *E. coli* per gram of feces). Enterocolitype 4 is characterized by the dominant and/or exclusive (>90%) presence of phylogroup B2, whereas enterocolitype 5 is characterized by the dominance of phylogenetic group A associated with various other phylogenetic groups. The abundance of strains from the four phylogenetic groups in these two human enterocolitypes is significantly different (*P *<* *0.001) (Fig.2, lower panel).

### Comparison of B2 commensal strains between humans and animals

Enterocolitype 1, which we identified in animals (low counts of *E. coli* with dominant B2 phylogroup), and enterocolitype 4, which we observed in humans (high counts of *E. coli* with dominant and/or exclusive B2 phylogroup) (Fig.2), differ in terms of the amount of B2 phylogroup strains that they contain (*P *<* *0.001); therefore, we analyzed more thoroughly the B2 commensal strains of humans and animals with the aim of identifying specific characteristics to differentiate these strains.

We studied 43 B2 phylogroup commensal strains (one per individual) isolated by plating either in this study in animals (13 strains) or in our previous study in humans (30 strains) (Smati et al. 2013). These 13 animal isolates were found in 11 wild animals and two domesticated animals and were always found with other phylogroups (“dominant non-exclusive” strains). In humans, 20 of the B2 strains belonged to the exclusive phylogroup (“exclusive” strains), whereas 10 strains were found in combination with other phylogroups (“dominant non-exclusive” strains). Consequently, we divided the B2 commensal strains into three categories: human “exclusive” strains, human “non-exclusive strains,” and animal “non-exclusive strains.” We used allele-specific PCR to assign the B2 strains to the main B2 subgroups (STc) (Clermont et al. 2014) and to determine their O-types (Clermont et al. 2007). We also analyzed their virulence factors and their ability to produce phages and colicins (Table S1).

**Figure 3:**
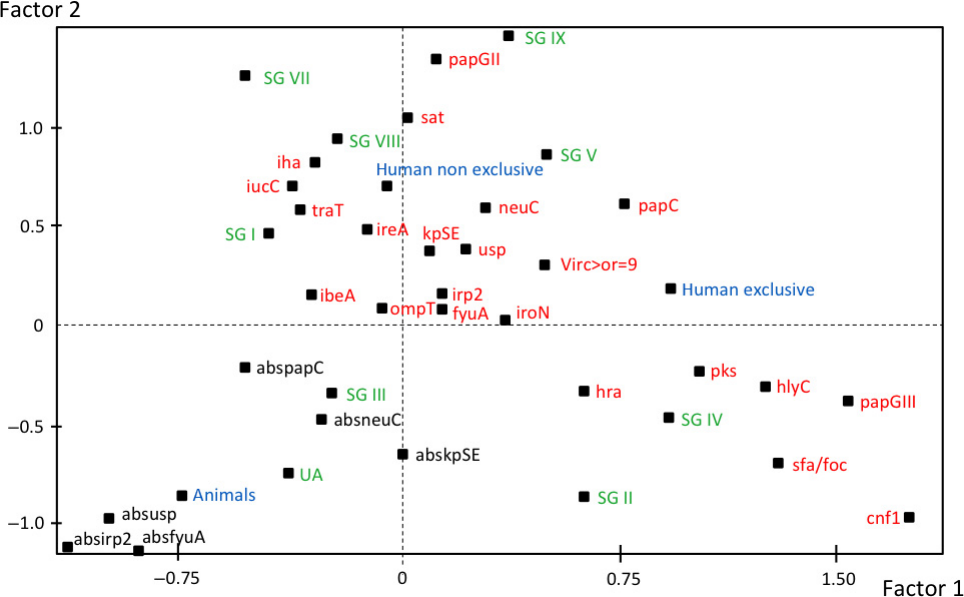
Factorial analysis of correspondence for the 43 phylogroup B2 *Escherichia coli* commensal strains of human or animal origin. Projections on the plane F1–F2 of the variables: human and “exclusive” B2 group, human and “non-exclusive” B2 group, animal origin (animals), the B2 subgroups (SG) I, II, III, IV, V, VII, VIII, and IX, unassigned (UA), the virulence factors described in Table3, the absence (abs) of several virulence factors, and a virulence score of 9 or more (Virc ≥ 9). The presence and absence of virulence genes are in red and black, respectively, the phylogenetic subgroups are in green and the origins of the strains are in blue.

We first described these data with a FAC (Fig.3). On the F1–F2 plane of the FAC, which accounted for 27.52% of the total variance, three groups of variables could be distinguished. The first group was characterized by the negative values of the two factors and included the animal origin, the absence of several virulence factors, the unassigned B2 subgroup, and the subgroup III (STc127 according to the Achtman multilocus sequence typing numbering scheme). The second group was characterized by the positive values of the second factor and comprised the human and “non-exclusive” B2 group strains, the B2 subgroups I (STc131), V (STc144), VII (STc14), VIII (STc452), and IX (STc95) strains, and the virulence factors *papGII*, *sat*, *iha*, *traT*, *ibeA*, *iuc*C, *ireA*, *neuC*, and *usp*. The third group was characterized by the positive values of the first factor and included the human and “exclusive” B2 group strains, the B2 subgroup II (STc73) and IV (STc141) strains, the virulence factors *cnf1*, *sfa/foc*, *papGIII*, *pks*, *hlyC*, *hra, iroN*, and *papC*, and a virulence score of 9 or more virulence factors. The O types were not projected on the FAC because there were too many of them, although the animal strains tended to be mostly nontypable with the panel of O-types that we tested, whereas the human strains belonged to classical O types found in extraintestinal strains (Clermont et al. 2007) (Table S1). The whole data set thus reflects strong differences between human and animal B2 strains and, to a lesser extent, between human “non-exclusive” and “exclusive” strains.

An analysis of the distribution of the 20 virulence factors in the three populations confirmed these findings (Table3). The median virulence factor score was 10.45 and 9.40 for “exclusive” and “non-exclusive” B2 human strains, respectively, whereas it was lower for animal isolates (6.38, *P *<* *0.001). Two virulence factors were significantly rarer in B2 animal strains than in B2 human strains: *papC*, which encodes pilus (*P *=* *0.004), and *usp*, which encodes uropathogenic-specific protein (*P *<* *0.001). Interestingly, the prevalence of two virulence factors differed significantly (*P *=* *0.05) between B2 “exclusive” human strains and other non-exclusive strains, irrespective of their human or animal origin. These factors were *sfa/foc*, which encode the F1C fimbriae (present in 50% of human “exclusive” isolates versus only 10% of human “non-exclusive” isolates and 15.38% of animal isolates) and *clbQ,* which is part of the *pks* island and encodes the genotoxin colibactin (present in 60% of human “exclusive” isolates versus only 20% of human “non-exclusive” isolates, and 15.38% of animal isolates). Colicin/phage production was similar between the three groups (data not shown).

**Table 3:** Prevalence of 20 virulence factors and virulence score of human and animal *Escherichia coli* B2 commensal strains

Genes	Description	Human “exclusive” (%) (*n *=* *20)	*P* value*	Human “non-exclusive” (%) (*n *=* *10)	Animal (%) (*n *=* *13)	*P* value*
*papC*	Pilus	45	0.005^a^	40	0	0.004^b^
*papGIII*	Pilus	15		10	0	
*sfa/foc*	F1C-fimbriae	50	0.06^a^	10	15.38	
			0.05^c^			
*iha*	Iron-regulated-gene-homolog adhesin	15		40	0	
*hra*	Heat-resistant agglutinin	35		10	38.46	
*ibeA*	Invasion of brain endothelium	40		40	46.15	
*fyuA*	Yersiniabactin receptor	95		90	84.62	
*irp2*	Yersiniabactin synthesis	95		90	84.62	
*iroN*	Salmochelin receptor	80		60	46.15	
*aer*	Aerobactin synthesis	40		20	7.69	
*ireA*	Iron-responsive element	20		40	30.77	
*kpsMTII*	Group II capsule antigen	60		70	61.54	
*neuC*	K1 capsular polysaccharide	60		50	23.08	
*ompT*	Outer membrane protein	80		90	84.62	
*traT*	Transfert protein	50		80	46.15	
*hlyC*	Haemolysin	35		20	7.69	
*cnf1*	Cytotoxic necrotizing factor 1	30		10	0	
*usp*	Uropathogenic-specific protein	95	0.002^a^	100	46.15	<0.001^b^
*sat*	Secreted autotransporter toxin	20		30	0	
*clbQ*	Genotoxin colibactin	60	0.01^a^	20	15.38	0.04^b^
			0.05^c^			
VFscore	As in Lefort et al. 2011, calculated on 20 VFs	10.45 ± 2.52		9.40 ± 2.67	6.38 ± 2.63	<0.001^b^

VF, virulence factor.

*a Significant *P*-value between human “exclusive” and animal isolates

b Significant *P*-value between human and animal isolates

c Significant *P*-value between human “exclusive” and human “non-exclusive” isolates.

## Discussion

Little is known about the genetic structure of populations of *E. coli* commensal strains; however, such data are indispensable for our understanding of the normal ecology of this major facultative anaerobic bacterium of the gut. This lack of data is mainly explained by the limitations of classically used quantitative methods, which rely on the study of colonies on plates, a tedious and restricted approach. We recently developed a quantitative PCR assay that can detect minor clones corresponding to up to 0.1% of the total *E. coli* population, and applied this technique to the human *E. coli* microbiota. In the current study, we extended this analysis to the *E. coli* microbiota of healthy animals living in the same region and at the same time as the humans included in our previous study. The microbiota of animals is more diverse than that of humans and includes additional *E. coli* phylogroups; nonetheless, our method is able to distinguish the main *E. coli* phylogroups and remains statistically relevant.

Ecological interactions within or between species determine the relative abundances of the different phyla. It has been recently proposed that three main enterotypes exist in the human microbiota (Arumugam et al. 2011). We extended this concept to *E. coli* by defining “enterocolitypes” that contain a particular abundance of one or several of the main *E. coli* phylogroups. We identified five enterocolitypes based on the data obtained in this study and the reanalysis of human data from a previous study (Smati et al. 2013): three in animals (B2 dominant, B1 dominant, and A dominant) and two in humans (exclusive B2 dominant and A dominant). Interestingly, the factors that determine these ecotypes also influence the total diversity of the microbiota, that is, the phylogeny of the host, diet, and domestication. The studied farm animals are characterized by numerous features that distinguish them from wild animals, in addition to the host decrease genetic diversity. First, the farm animals were very young when their ages at sampling were compared to their theoretical lifetimes. Second, the population density was much lower in wild animals. Thus, in the forest of Fontainebleau, the density of deer and boars is about 4 and 8 individuals per 100 hectares, respectively (Jean Marc Cacouault, Office National des Forêts, pers. comm.), whereas the farm animals that have been studied are living in crowded conditions (50 cows in a few hundred square meters to 1000 chicken in 100 m^2^). Lastly, the diet is also different with the use of antibiotics in the farm animals. All these features may contribute to the observed differences in the microbiota between domesticated and wild animals. Altogether, these data indicate that a complex network of interactions may exist within the intestinal microbiota between the various genera and phylogenetic groups of the same species (Trosvik et al. 2015). This network probably involves the availability of various nutrients coupled to the specific metabolism of bacteria, cross-feeding and toxic interactions involving phages and/or colicins. Of note, we did not identify any differences in the production of phages and colicin between the various B2 strains.

B2 phylogroup strains differ from most other *E. coli* strains because they are involved in extraintestinal infections, but are also present as commensals (Nowrouzian et al. 2005). It has been proposed that virulence is a by-product of commensalism (Le Gall et al. 2007; Diard et al. 2010). Our study provides further insight into B2 strains. Indeed, we show that (1) B2 clones isolated from humans correspond mainly to classical extraintestinal pathogenic clones including subgroups I (STc131), II (STc73), and IX (STc 95) (Clermont et al. 2014), whereas those isolated from animals correspond to a much wider range of subgroups (six of 13 strains were unassignable using our targeted subgroup characterization) (Table S1); and (2) “exclusive” dominant B2 strains in humans are characterized by two specific virulence factor, *sfa/foc* and *pks*. The gene *sfa/foc*, which encodes a subunit of F1C fimbriae, may enable adherence to intestinal porcine epithelial cells (Schierack et al. 2013). The *E. coli* Nissle 1917 strain, a robust colonizer of the human gastrointestinal tract, requires F1C fimbriae to make biofilms. In addition, these structures are also important for colonization in newborn mice (Lasaro et al. 2009). Long-term *E. coli* colonizers were also significantly more likely to have the *pks* island than transient strains, suggesting that the *pks* island is partly responsible for the strong gut-colonizing capacity of group B2 strains (Nowrouzian and Oswald 2012). This effect may result from the genotoxicity of the *pks*-derived compounds that slow the renewal of the intestinal epithelium by blocking the cell cycle (Nougayrède et al. 2006).

The limitations of our study are of two types. First, we sampled farm animals in one farm for each species and our results need to be confirmed on more animals from several farms. Second, we do not quantify the diversity of the clones within a single phylogroup and it has been established that *E. coli* populations can consist of many clones in human (Schlager et al. 2002; Moreno et al. 2009) and animals (Schierack et al. 2008). This last point will be resolved in the future by developing quantitative genomic assays based on high throughput sequencing to quantify *E. coli* abundance at the clone level. Despite these limitations, our work is the first one to have studied quantitatively the *E. coli* microbiota diversity in a wide range of wild and domesticated animals.

In conclusion, our findings, in addition to those of previous studies (Ley et al. 2008; Arumugam et al. 2011; Wu et al. 2011; Smati et al. 2013), show that a complex network of interactions exists at several levels within the intestinal microbiota, including interactions between genera and within species, as exemplified by the most abundant aerobic facultative species of the gut, *E. coli*. These interactions result in specific combinations characterized by a particular abundance of different phyla, either at the genus and intra-species levels. Interestingly, the forces that shape the enterotype structure at the genus level as the phylogeny of the host, diet, domestication (Muegge et al. 2011; Wu et al. 2011; David et al. 2014) also affect the entercolitype structure at the *E. coli* species level.

## Conflict of Interest

None declared.

## Supporting Information

**Table S1.** Main genotypic characteristics of the 43 phylogroup B2 *Escherichia coli* commensal strains of human or animal origin.
